# Supplementary material for: Integrated Transcriptomics and Metabolomics Analysis of the Fructan Metabolism Response to Low-Temperature Stress in Garlic
Source: Genes (Basel). 2023 Jun 19;14(6):1290. doi: 10.3390/genes14061290 (PMC10298409; doi:10.3390/genes14061290)
Supplement: Supplementary file 1 [file genes-14-01290-s001.zip › supplementary materials/Table S1-S3.pdf]

Table S1:Statistical table of sequencing output

Sample	Raw Reads	Clean Reads	Clean Base (G)	Error Rate (%)	Q20 (%)	Q30 (%)	GC Content (%)
TCK01	42913636	40672752	6.1	0.02	98.44	95.29	44.65
TCK02	42938924	40748638	6.11	0.02	98.43	95.3	44.29
TCK03	44752492	42209476	6.33	0.02	98.49	95.44	43.81
TDT51	44379416	42222758	6.33	0.02	98.4	95.1	44.94
TDT52	43861306	40905182	6.14	0.02	98.39	95.19	44.47
TDT53	45355302	43820382	6.57	0.02	98.57	95.52	44

Table S2:Statistical table of assembly results

Type	Number	Mean Length	N50	N90	Total Bases
Transcript	406864	921	1426	393	374833155
Unigene	328951	1073	1517	501	353004383

Table S3:Statistical table of assembly results

Database	Number of Genes	Percentage (%)
KEGG	118659	36.07
NR	149917	45.57
SwissProt	107033	32.54
Trembl	148075	45.01
KOG	92903	28.24
GO	120931	36.76
Pfam	106551	32.39
Annotated in at least one Database	152066	46.23
Total Unigenes	328951	100
