# Supplementary figures and images for: Integrated Transcriptomics and Metabolomics Analysis of the Fructan Metabolism Response to Low-Temperature Stress in Garlic
Source: Genes (Basel). 2023 Jun 19;14(6):1290. doi: 10.3390/genes14061290 (PMC10298409; doi:10.3390/genes14061290)

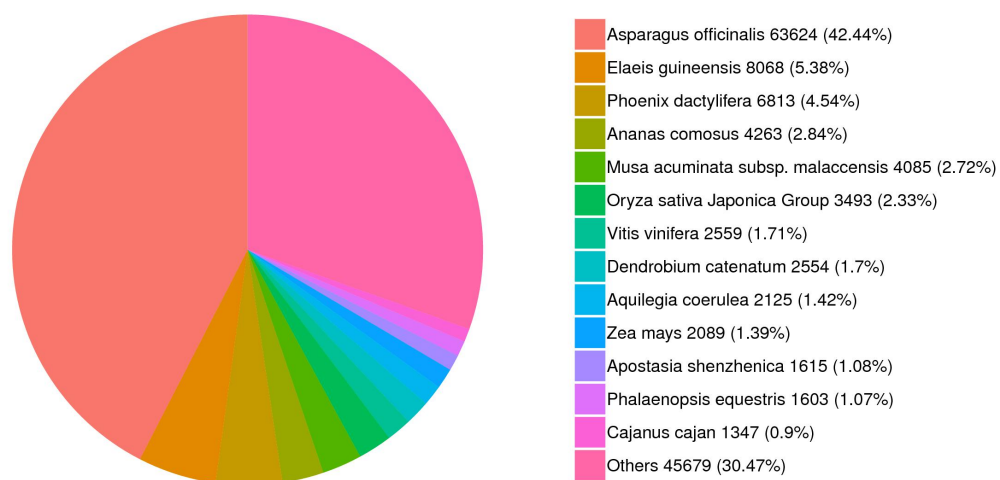

**Supplemental Figure S1 : NR Annotation Pie Chart.**

Supplement: Supplementary file 1 [file genes-14-01290-s001.zip › supplementary materials/Figure S1.pdf]
